# Supplementary material for: WS2 Nanosheet Loaded Silicon-Oxycarbide Electrode for Sodium and Potassium Batteries
Source: Nanomaterials (Basel). 2022 Nov 25;12(23):4185. doi: 10.3390/nano12234185 (PMC9736738; doi:10.3390/nano12234185)
Supplement: Supplementary file 1 [file nanomaterials-12-04185-s001.zip › nanomaterials-1996528-supplementary.pdf]

Supplementary Materials

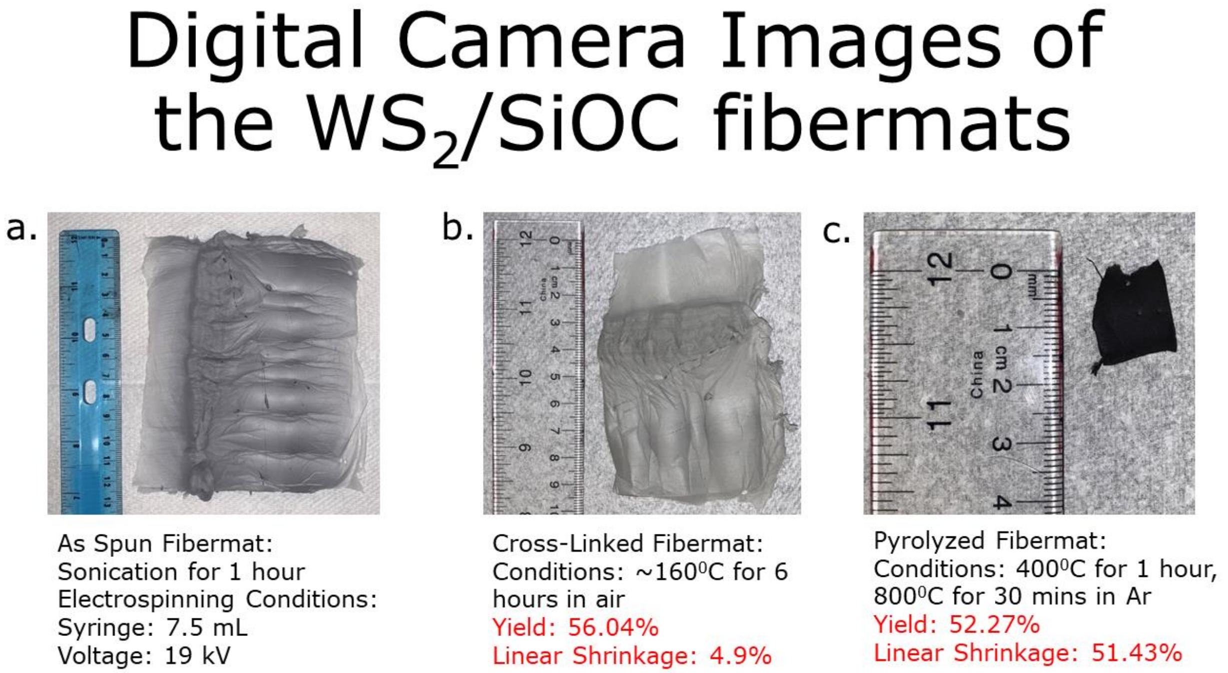

Figure S1: Digital camera images of the WS_2/SiOC composite fibermats in (**a**) as-spun; (**b**) cross-linked; (**c**) pyrolyzed forms with corresponding weight retention and linear shrinkage values.

Table S1: Elemental Composition of the fibermats by XPS

Pyrolyzed Fibermats	Elements (Atomic %)				
	W4f	S2p	Si2p	C1s	O1s
WS_2/SiOC	1.50367	1.19286	9.56574	72.4238	15.3139

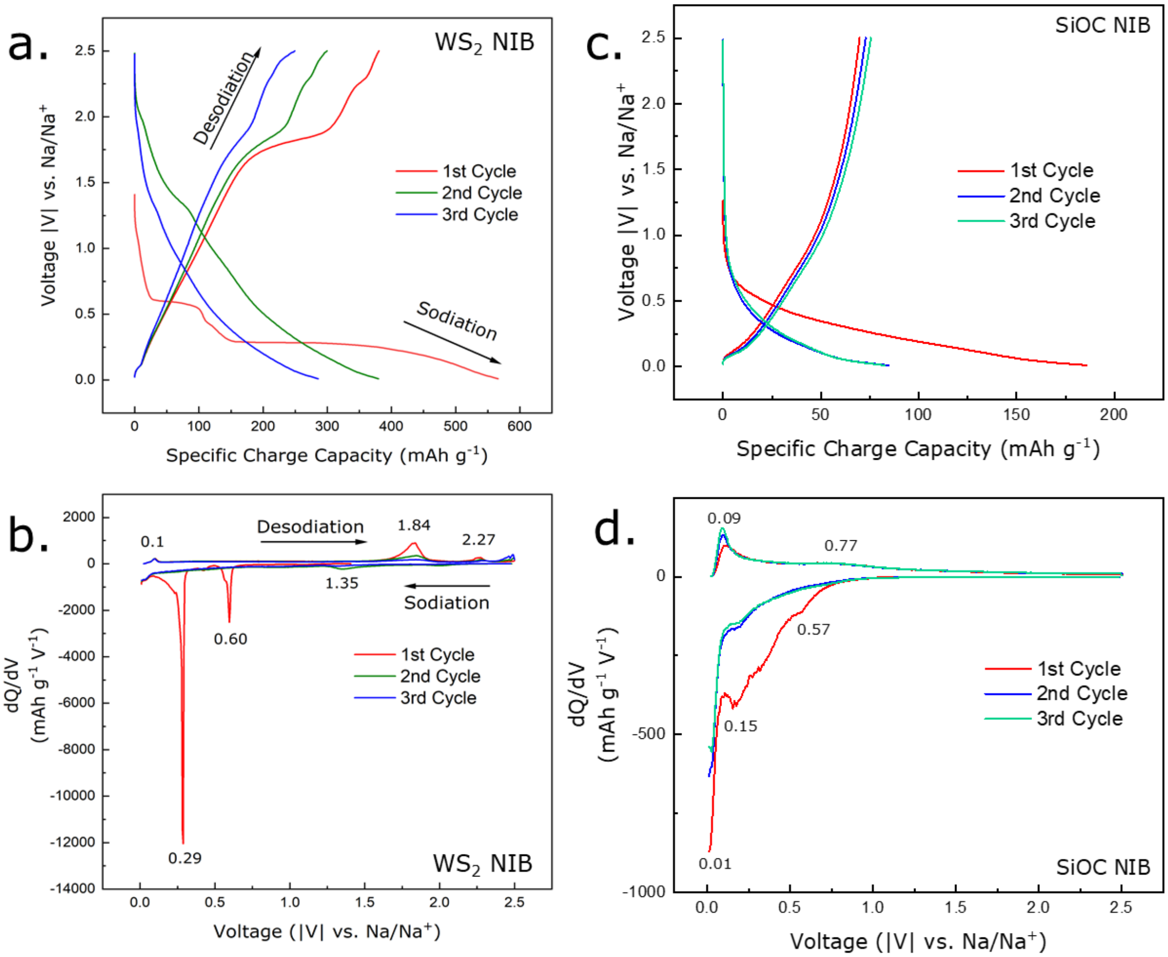

Figure S2: (**a**) GCD profile of the WS_2 neat electrode when tested in a Na^+ ion half-cell setup; (**b**) differential capacity curve of the WS_2 neat electrode in Na^+ ion half-cell setup derived from the GCD profile providing information regarding reactions taking place at different voltages; (**c**) GCD profile of the SiOC fibermat electrode when tested in a Na^+ ion half-cell setup; (**d**) differential capacity curve of the SiOC fibermat electrode in Na^+ ion half-cell setup derived from the GCD profile providing information regarding reactions taking place at different voltages.

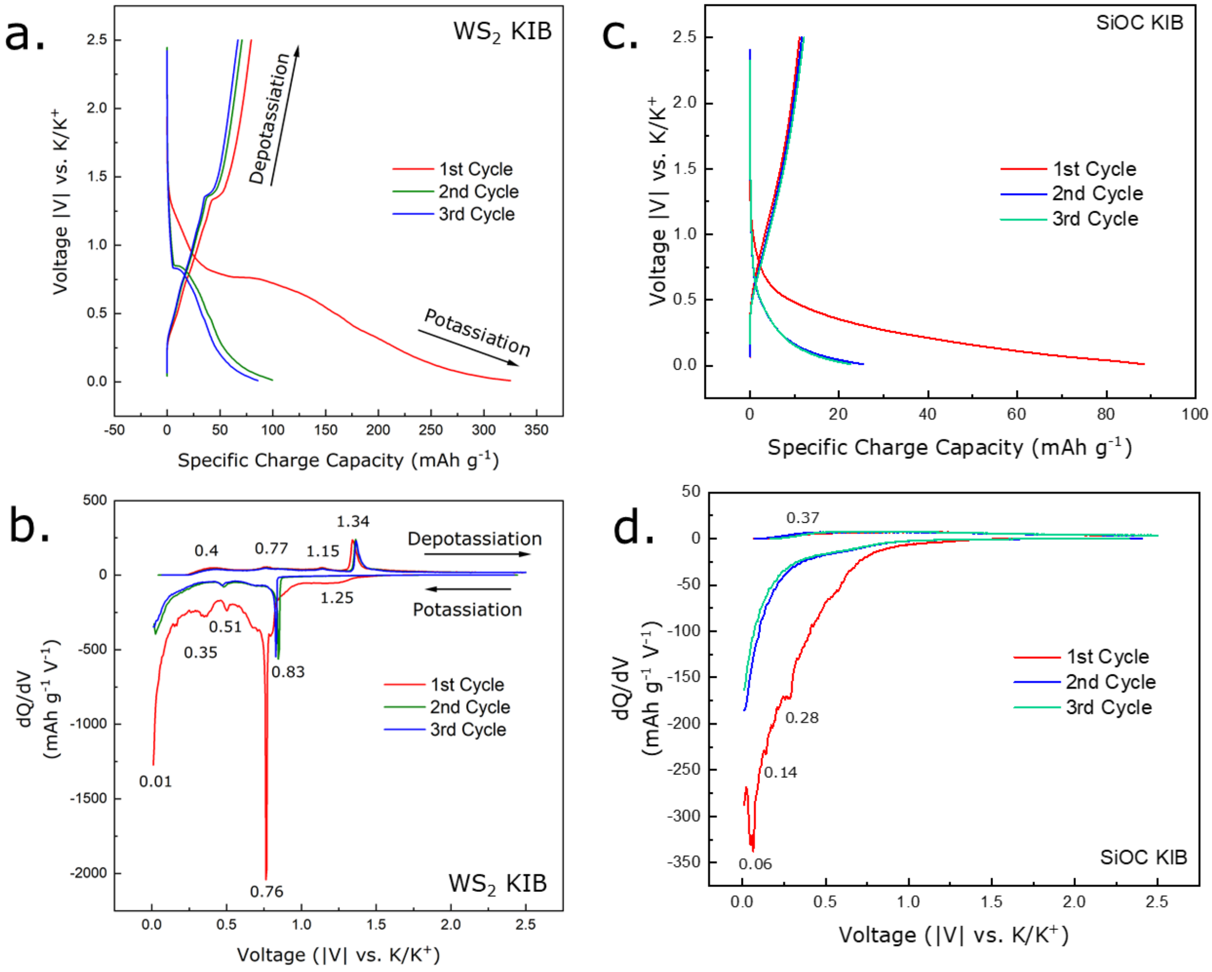

Figure S3: (**a**) GCD profile of the WS_2 neat electrode when tested in a K^+ ion half-cell setup; (**b**) differential capacity curve of the WS_2 neat electrode in K^+ ion half-cell setup derived from the GCD profile providing information regarding reactions taking place at different voltages; (**c**) GCD profile of the SiOC fibermat electrode when tested in a K^+ ion half-cell setup; (**d**) differential capacity curve of the SiOC fibermat electrode in K^+ ion half-cell setup derived from the GCD profile providing information regarding reactions taking place at different voltages.

Table S2: Summary and comparison of electrochemical performance for Na^+ storage of other nanomaterial-based structures with this work.

**Anode Materials**	**Rate Capability**	**Reference**
WS_2/SiOC	474.88 mAh g^{-1} @100 mA g^{-1}	This work
	399.68 mAh g^{-1} @200 mA g^{-1}	
	313.33 mAh g^{-1} @400 mA g^{-1}	
$W_{0.9}Mo_{0.1}S_2$	262 mAh g^{-1} @1000 mA g^{-1}	[1]
WS_2/NC	320 mAh g^{-1} @200 mA g^{-1}	[2]
MoS_2/RGO	253.1 mAh g^{-1} @100 mA g^{-1}	[3]
WS_2-S/N-C	319 mAh g^{-1} @100 mA g^{-1}	[4]
MoS_2-N-RGO	250 mAh g^{-1} @1000 mA g^{-1}	[5]
WS_2/3DCD	392.1 mAh g^{-1} @200 mA g^{-1}	[6]
DODA-WS_2	318 mAh g^{-1} @1000 mA g^{-1}	[7]
1T-MoS_2	324 mAh g^{-1} @1000 mA g^{-1}	[8]
2H-WS_2	353.2 mAh g^{-1} @200 mA g^{-1}	[9]
WS_2@moS_2@C/rGO	411.8 mAh g^{-1} @500 mA g^{-1}	[10]
WS_2/CNT-rGO ordered 3D aerogel	311.4 mAh g^{-1} @100 mA g^{-1}	[11]
	302.8 mAh g^{-1} @200 mA g^{-1}	
	289 mAh g^{-1} @500 mA g^{-1}	
MoS_2/graphene paper	240 mAh g^{-1} @25 mA g^{-1}	[12]
	214 mAh g^{-1} @100 mA g^{-1}	
	173 mAh g^{-1} @200 mA g^{-1}	
MXene@Co_9S_8/$CoMo_2S_4$	325 mAh g^{-1} @100 mA g^{-1}	[13]
	309 mAh g^{-1} @200 mA g^{-1}	
	284 mAh g^{-1} @100 mA g^{-1}	

Table S3: Summary and comparison of electrochemical performance for K^+ storage of other nanomaterial-based structures with this work.

**Anode Materials**	**Rate Capability**	**Reference**
WS_2/SiOC	218.91 mAh g^{-1} @100 mA g^{-1}	This work
	158.16 mAh g^{-1} @200 mA g^{-1}	
	125.92 mAh g^{-1} @400 mA g^{-1}	
D-TiS_2	124 mAh g^{-1} @50 mA g^{-1}	[14]
	100 mAh g^{-1} @100 mA g^{-1}	
$TiSe_2$	89 mAh g^{-1} @50 mA g^{-1}	[15]
	67 mAh g^{-1} @100 mA g^{-1}	
Commercial WS_2	109 mAh g^{-1} @50 mA g^{-1}	[16]
	74 mAh g^{-1} @100 mA g^{-1}	
Hexagonal 2H-WS_2	67 mAh g^{-1} @5 mA g^{-1}	[17]
	40 mAh g^{-1} @200 mA g^{-1}	
$HeTiO_{2e}C$ Micro-tubes	197.5 mAh g^{-1} @100 mA g^{-1}	[18]
Graphite	197 mAh g^{-1} @C/2	[19]
Soft Carbon	160 mAh g^{-1} @2C	[19]
Hard Carbon Microspheres	216 mAh g^{-1} @C/10	[20]
Tin Based Composite	110 mAh g^{-1} @25 mA g^{-1}	[21]
Reduced graphene oxide	90 mAh g^{-1} @50 mA g^{-1}	[22]
	50 mAh g^{-1} @100 mA g^{-1}	
Tire -derived Carbon	155 mAh g^{-1} @140 mA g^{-1}	[23]

References

1. Tao, H.; Li, J.; Li, J.; Hou, Z.; Yang, X.; Fan, L.-Z. Metallic phase W0.9Mo0.1S2 for high-performance anode of sodium ion batteries through suppressing the dissolution of polysulfides. *Journal of Energy Chemistry* **2022**, *66*, 356-365, doi:https://doi.org/10.1016/j.jechem.2021.08.026.
2. Lim, Y.V.; Wang, Y.; Kong, D.; Guo, L.; Wong, J.I.; Ang, L.K.; Yang, H.Y. Cubic-shaped WS2 nanopetals on a Prussian blue derived nitrogen-doped carbon nanoporous framework for high performance sodium-ion batteries. *Journal of Materials Chemistry A* **2017**, *5*, 10406-10415, doi:10.1039/C7TA01821E.
3. Zhang, X.; Liu, K.; Zhang, S.; Miao, F.; Xiao, W.; Shen, Y.; Zhang, P.; Wang, Z.; Shao, G. Enabling remarkable cycling performance of high-loading MoS2@Graphene anode for sodium ion batteries with tunable cut-off voltage. *Journal of Power Sources* **2020**, *458*, 228040, doi:https://doi.org/10.1016/j.jpowsour.2020.228040.
4. Li, X.; Sun, Y.; Xu, X.; Wang, Y.-X.; Chou, S.-L.; Cao, A.; Chen, L.; Dou, S.-X. Lotus rhizome-like S/N–C with embedded WS2 for superior sodium storage. *Journal of Materials Chemistry A* **2019**, *7*, 25932-25943, doi:10.1039/C9TA09373G.
5. Zhan, W.; Zhu, M.; Lan, J.; Yuan, H.; Wang, H.; Yang, X.; Sui, G. All-in-One MoS2 Nanosheets Tailored by Porous Nitrogen-Doped Graphene for Fast and Highly Reversible Sodium Storage. *ACS Applied Materials & Interfaces* **2020**, *12*, 51488-51498, doi:10.1021/acsami.0c15169.
6. Wang, Y.; Kong, D.; Huang, S.; Shi, Y.; Ding, M.; Von Lim, Y.; Xu, T.; Chen, F.; Li, X.; Yang, H.Y. 3D carbon foam-supported WS2 nanosheets for cable-shaped flexible sodium ion batteries. *Journal of Materials Chemistry A* **2018**, *6*, 10813-10824, doi:10.1039/C8TA02773K.
7. Xu, X.; Li, X.; Zhang, J.; Qiao, K.; Han, D.; Wei, S.; Xing, W.; Yan, Z. Surfactant assisted electrospinning of WS2 nanofibers and its promising performance as anode material of sodium-ion batteries. *Electrochimica Acta* **2019**, *302*, 259-269, doi:https://doi.org/10.1016/j.electacta.2019.02.042.
8. Sun, D.; Huang, D.; Wang, H.; Xu, G.-L.; Zhang, X.; Zhang, R.; Tang, Y.; Abd Ei-Hady, D.; Alshitari, W.; Saad Al-Bogami, A.; et al. 1T MoS2 nanosheets with extraordinary sodium storage properties via thermal-driven ion intercalation assisted exfoliation of bulky MoS2. *Nano Energy* **2019**, *61*, 361-369, doi:https://doi.org/10.1016/j.nanoen.2019.04.063.
9. Luo, X.; Huang, J.; Huang, Y.; Cao, L.; Li, J.; Wang, Y.; Xu, Z.; Wei, S.; Kajiyoshi, K. Self-templated induced carbon-supported hollow WS2 composite structure for high-performance sodium storage. *Journal of Materials Chemistry A* **2021**, *9*, 21366-21378, doi:10.1039/D1TA04858A.
10. Rao, Y.; Wang, J.; Liang, P.; Zheng, H.; Wu, M.; Chen, J.; Shi, F.; Yan, K.; Liu, J.; Bian, K.; et al. Heterostructured WS2/MoS2@carbon hollow microspheres anchored on graphene for high-performance Li/Na storage. *Chemical Engineering Journal* **2022**, *443*, 136080, doi:https://doi.org/10.1016/j.cej.2022.136080.
11. Wang, Y.; Kong, D.; Shi, W.; Liu, B.; Sim, G.J.; Ge, Q.; Yang, H.Y. Ice Templated Free-Standing Hierarchically WS2/CNT-rGO Aerogel for High-Performance Rechargeable Lithium and Sodium Ion Batteries. *Advanced Energy Materials* **2016**, *6*, 1601057, doi:https://doi.org/10.1002/aenm.201601057.
12. David, L.; Bhandavat, R.; Singh, G. MoS2/Graphene Composite Paper for Sodium-Ion Battery Electrodes. *ACS Nano* **2014**, *8*, 1759-1770, doi:10.1021/nn406156b.
13. Wang, M.; Liu, X.; Qin, B.; Li, Z.; Zhang, Y.; Yang, W.; Fan, H. In-situ etching and ion exchange induced 2D-2D MXene@Co9S8/CoMo2S4 heterostructure for superior Na+ storage. *Chemical Engineering Journal* **2023**, *451*, 138508, doi:https://doi.org/10.1016/j.cej.2022.138508.
14. Liu, T.; Zhang, X.; Xia, M.; Yu, H.; Peng, N.; Jiang, C.; Shui, M.; Xie, Y.; Yi, T.-F.; Shu, J. Functional cation defects engineering in TiS2 for high-stability anode. *Nano Energy* **2020**, *67*, 104295, doi:https://doi.org/10.1016/j.nanoen.2019.104295.
15. Li, P.; Zheng, X.; Yu, H.; Zhao, G.; Shu, J.; Xu, X.; Sun, W.; Dou, S.X. Electrochemical potassium/lithium-ion intercalation into TiSe2: Kinetics and mechanism. *Energy Storage Materials* **2019**, *16*, 512-518, doi:https://doi.org/10.1016/j.ensm.2018.09.014.
16. Wu, Y.; Xu, Y.; Li, Y.; Lyu, P.; Wen, J.; Zhang, C.; Zhou, M.; Fang, Y.; Zhao, H.; Kaiser, U.; et al. Unexpected intercalation-dominated potassium storage in WS2 as a potassium-ion battery anode. *Nano Research* **2019**, *12*, 2997-3002, doi:10.1007/s12274-019-2543-0.
17. Zhang, R.; Bao, J.; Pan, Y.; Sun, C.-F. Highly reversible potassium-ion intercalation in tungsten disulfide. *Chemical Science* **2019**, *10*, 2604-2612, doi:10.1039/C8SC04350G.
18. Li, Y.; Yang, C.; Zheng, F.; Pan, Q.; Liu, Y.; Wang, G.; Liu, T.; Hu, J.; Liu, M. Design of TiO2eC hierarchical tubular heterostructures for high performance potassium ion batteries. *Nano Energy* **2019**, *59*, 582-590, doi:https://doi.org/10.1016/j.nanoen.2019.03.002.
19. Jian, Z.; Luo, W.; Ji, X. Carbon Electrodes for K-Ion Batteries. *Journal of the American Chemical Society* **2015**, *137*, 11566-11569, doi:10.1021/jacs.5b06809.
20. Jian, Z.; Xing, Z.; Bommier, C.; Li, Z.; Ji, X. Hard Carbon Microspheres: Potassium-Ion Anode Versus Sodium-Ion Anode. *Advanced Energy Materials* **2016**, *6*, 1501874, doi:https://doi.org/10.1002/aenm.201501874.
21. Sultana, I.; Ramireddy, T.; Rahman, M.M.; Chen, Y.; Glushenkov, A.M. Tin-based composite anodes for potassium-ion batteries. *Chemical Communications* **2016**, *52*, 9279-9282, doi:10.1039/C6CC03649J.

22. Luo, W.; Wan, J.; Ozdemir, B.; Bao, W.; Chen, Y.; Dai, J.; Lin, H.; Xu, Y.; Gu, F.; Barone, V.; et al. Potassium Ion Batteries with Graphitic Materials. *Nano Letters* **2015**, *15*, 7671-7677, doi:10.1021/acs.nanolett.5b03667.

23. Li, Y.; Adams, R.A.; Arora, A.; Pol, V.G.; Levine, A.M.; Lee, R.J.; Akato, K.; Naskar, A.K.; Paranthaman, M.P. Sustainable Potassium-Ion Battery Anodes Derived from Waste-Tire Rubber. *Journal of The Electrochemical Society* **2017**, *164*, A1234, doi:10.1149/2.1391706jes.
